# Construction of an engineered *Bacillus subtilis* for production of poly-γ-glutamic acids with specific molecular weights

**DOI:** 10.3389/fmicb.2025.1597704

**Published:** 2025-09-03

**Authors:** Yu Lin, Yiwei Ding, Lin Shu, Huizhen Chen, Nan Li, Xiaoqun Duan, Wei Zeng

**Affiliations:** ^1^Key Laboratory of Molecular Medical Engineering, Education Department of Guangxi Zhuang Autonomous Region, School of Intelligent Medicine and Biotechnology, Guilin Medical University, Guilin, China; ^2^College of Life Science and Technology, Guangxi University, Nanning, China; ^3^School of Biomedical Industry, Guilin Medical University, Guilin, China

**Keywords:** poly-γ-glutamic acid, molecular weight, *Bacillus subtilis*, hydrolase, dynamic regulation

## Abstract

**Introduction:**

Poly-γ-glutamic acid (γ-PGA) with different molecular weight (Mw) exhibits different properties and therefore has a variety of applications. At present, the γ-PGA is mainly produced by *Bacillus* species. However, the production of γ-PGAs with specific Mws often requires multiple strains, which limits the development and application of γ-PGA.

**Methods:**

To address this limitation, we constructed an engineered *Bacillus subtilis* strain by deleting hydrolase genes *cwlO*, *pgdS* and *ggt*, and further introduced regulation of PgdS expression under an IPTG-inducible promoter.

**Results:**

When the hydrolase genes *cwlO*, *pgdS* and *ggt* in *B. subtilis* were deleted, the γ-PGA Mw and titer increased by 220.1% (2.42×10^7^ Da) and 47.81% (8.44 g/L), respectively. Furthermore, regulation of PgdS expression enabled dynamic control of γ-PGA Mw. The γ-PGA with Mw ranging from 9.55×10^4^ Da to 2.15×10^7^ Da was produced by change of IPTG addition time in an engineered strain, with the titer of 6.28-8.57 g/L. In the 5-L fermenter, the γ-PGA with Mw ranging from 8.3×10^4^ Da to 1.87×10^7^ Da was produced under optimal conditions.

**Conclusion:**

In summary, an engineered strain that can dynamically regulate the γ-PGA Mw and produce γ-PGAs with specific Mws was obtained, and its regulatory range was wider than that of previous studies, which increased the application potential.

## 1 Introduction

Poly-γ-glutamic acid (γ-PGA) is a natural polymer composed of repeating units of D-and/or L-glutamic acid linked via γ-amide bonds (Ashiuchi and Misono, 2002), and is mainly synthesized in *Bacillus* species, including *Bacillus subtilis*, *Bacillus licheniformis*, and *Bacillus amyloliquefaciens* (Hsueh et al., 2017). Due to its nontoxicity, compatibility, and biodegradability, it has been used in variety fields, including food, cosmetics, medicine and agriculture (Wang et al., 2022). It is worth noting that the molecular weight (Mw) of γ-PGA plays an important role in its application. For example, γ-PGA with low-Mw (<400 kDa) can be used as a material for the production of hydrogel sunscreens without irritation and sensitization (Wang et al., 2019). It can also be applied as drug carriers to control drug release (Balogun-Agbaje et al., 2021). γ-PGA with high-Mw (>400 kDa) can remove heavy metals or dye from wastewater (Mark et al., 2006; Stephen Inbaraj et al., 2006). γ-PGA with ultra-high-Mw (>4,000 kDa) can be used as a fertilizer synergist to decrease the use of chemical fertilizer and improve crop yield (Bai et al., 2020). Thus, achievement of production of γ-PGAs with different Mws is critical to the development and application of γ-PGAs.

Traditionally, the regulation of γ-PGA Mw was achieved by controlling fermentation conditions, such as culture components and conditions (Jung et al., 2005; Zeng et al., 2014; Feng et al., 2017). However, these changes were specific for each strain. Moreover, the γ-PGA Mw cannot be precisely regulated. In contrast, it is more effective to modify strains by molecular biology methods to improve the production of γ-PGA. At present, two types of enzymes have been identified that can degrade γ-PGA: the endo-type hydrolase and the exo-type hydrolase. The endo-type hydrolase cleaves the γ-amide bond within γ-PGA, fragmenting the polymer and reducing its Mw. This includes PgdS, which is secreted extracellularly to degrade γ-PGA (Cao et al., 2018). The exo-type hydrolase can degrade γ-PGA to release glutamic acid monomers, including CwlO and GGT (Kimura et al., 2004; Feng et al., 2014). The γ-PGA hydrolase activity was related to Mw and titer of γ-PGA. For instance, when the genes encoding PgdS and CwlO were deleted in *B. amyloliquefaciens*, the γ-PGA Mw increased from 3.28 × 10^5^ to 4.14 × 10^5^ Da, and titer increased by 93% (Feng et al., 2014). Besides, several engineered *Bacillus* strains were constructed to express different γ-PGA synthetases, resulting in production of γ-PGA with wide Mw range from 4 × 10^4^ to 8.5 × 10^6^ Da (Halmschlag et al., 2019). γ-PGAs with Mw ranging from 6.82 × 10^4^ to 1.78 × 10^6^ Da were produced by regulation of expression and secretion of PgdS in engineered *B. licheniformis* strains (Wang et al., 2020). However, in the above reports, a single strain typically produces γ-PGA with one specific Mw, which limits the potential application of γ-PGA.

Therefore, production of γ-PGAs with specific Mws by one-step has attracted much attention. For instance, Clustered Regularly Interspaced Short Palindromic Repeats interference (CRISPRi) system was introduced in *B. amyloliquefaciens* NBCSO-3 (Δ*pgdS*Δ*cwlO*) for dynamic regulation of PgdS expression level. γ-PGA in the Mw range of 5 × 10^4^–1.4 × 10^6^ Da was produced by an engineered strain (Sha et al., 2020b). However, given the outstanding potential of γ-PGA for industrial applications, the regulatory range of γ-PGA Mw deserves to be further broadened.

In this study, the expression of γ-PGA synthetase was controlled in *B. subtilis* 168 by promoter engineering. Furthermore, to produce ultra-high-Mw γ-PGA, the coding sequences of multiple γ-PGA hydrolases were deleted, and the γ-PGA Mw and titer in mutant strains were evaluated. Next, the γ-PGAs with specific Mws were produced by regulation of PgdS expression level in an engineered strain. Finally, the γ-PGA production process was scaled-up by 70-fold by conducting experiments at the 3.5-L culture volume in a bioreactor. This study aims to tailor production of γ-PGA and improve its potential applications.

## 2 Materials and methods

### 2.1 Strains, media, and culture conditions

*E. coli* DH5α was used for plasmid propagation and construction. *B. subtilis* 168 was used for production of γ-PGA.

The media included Luria-Bertani (LB) medium (5 g/L yeast extract, 10 g/L tryptone and 10 g/L NaCl) and fermentation medium (30 g/L glucose, 2.5 g/L yeast extract, 20 g/L L-glutamate, 1 g/L K_2_HPO_4_, 1 g/L MgSO_4_, pH 7.0).

All *E. coli* strains were cultured in LB medium at 37 °C. For γ-PGA production, an individual colony of *B. subtilis* strain was inoculated into 5 mL of LB medium at 37 °C overnight for seed culture preparations. Then, 1 mL of seed culture was inoculated into 50 mL of fermentation medium, which was incubated at 37 °C with shaking at 160 rpm for 48 h. For engineering *Bacillus* strains harboring the promoter P_xylA_, 4 g/L xylose was added to the cultures at 8 h, followed by continued incubation for an additional 40 h. Biomass was monitored by measuring the optical density at 660 nm (OD₆₆₀). The shake flask experiments were performed in triplicate.

For colony morphology analysis, fermentation medium agar plates were supplemented with 4 g/L xylose and 2% agar. *B. subtilis* strains were streaked on the plates and incubated at 37 °C for 24 h.

In the carbon source optimization experiments, glucose was individually replaced with 30 g/L of sucrose, fructose, lactose, mannitol, or glycerol, respectively, while all other components of the fermentation medium remained unchanged. Xylose (4 g/L) was added to the cultures at 8 h, followed by continued incubation for an additional 40 h. In xylose concentration optimization experiments, xylose was added to the cultures at concentrations of 1, 2, 4, 8, and 16 g/L at 8 h, followed by continued incubation for an additional 40 h. All experiments were conducted in triplicate.

In the IPTG concentration optimization experiments, IPTG was added at concentrations of 0.1, 0.2, 0.4, 0.6, 0.8, and 1.0 mM to the cultures at 8 h, followed by an additional 8 h of incubation. To determine the optimal IPTG addition time point, 0.6 mM IPTG was added to the cultures at 0, 4, 8, 16, 20, and 24 h, respectively, followed by an additional 8 h of incubation. The activity of PgdS in the culture supernatant was measured to evaluate the optimal IPTG concentration and addition time point. All experiments were conducted in triplicate.

For scale-up, the cell grew in 5-L fermenter with 3.5 L of media (30 g/L mannitol, 2.5 g/L yeast extract, 20 g/L L-glutamate, 1 g/L K_2_HPO_4_, 1 g/L MgSO_4_, 0.04% (v/v) antifoam (THIX-298), pH 7.0) for 72 h. The inoculation amount and temperature were 2% (v/v) and 37 °C, respectively. The aeration rate was 1.0 vvm, and the agitation speed was 400 rpm. The biomass, γ-PGA titer, concentrations of mannitol and L-glutamate, Mw of γ-PGA were analyzed.

To further improve the γ-PGA production in 5-L fermenter, the effects of inoculation amount (1% (v/v), 2, 5 and 8%), temperature (32 °C, 37 °C, and 42 °C), aeration rate (0.5 vvm, 1.0 vvm, and 1.5 vvm), agitation speed (200 rpm, 300 rpm, 400 rpm, and 500 rpm) and pH (6.0, 6.5, 7.0, and pH-uncontrolled) were investigated.

### 2.2 Construction of plasmids and strains

The primers used for plasmid construction are shown in Supplementary Table S1. The sequences of promoter P_aprE_, P_43_, and P_xylA_ were amplified from the genomic DNA of *B. subtilis* 168. Two methods were used to construct the plasmid. One was to digest DNA fragments and plasmids with restriction enzymes, respectively, followed by ligation using T4 DNA ligase (Takara, Dalian). Another method was to insert DNA fragments into plasmids using an ClonExpress Ultra One Step Cloning Kit (Vazyme, China).

For expression of the *pgsBCAE* gene cluster, the upstream (1.5 kb) and downstream (1.5 kb) regions of *pgsB* were PCR-amplified from the genomic DNA of *B. subtilis* 168. These fragments were individually assembled with three different promoter sequences via overlapping PCR. The resulting constructs were then cloned into the plasmid pKSV7, yielding plasmids pKSV-*P_43_*, pKSV-*P_aprE_*, and pKSV-*P_xylA_*, respectively.

For genes *cwlO*, *pgdS* and *ggt* deletion, the upstream and downstream fragments regions of each target gene were amplified by PCR and fused by overlapping PCR. The fused fragments were subsequently inserted into plasmid pKSV7, generating plasmids pKSV*-cwlO*, pKSV-*pgdS*, and pKSV-*ggt*, respectively.

For *pgdS* expression, the coding sequence was amplified by PCR from *B. subtilis* 168 genomic DNA and inserted into plasmid pHT43, producing plasmid pHT-*SP_PgdS_*-*pgdS*. The sequences of signal peptide SP_amyQ_ and SP_aprE_ were PCR-amplified from pHT43 and pBE-S, respectively, and replaced the SP_PgdS_ sequence in plasmid pHT-*SP_PgdS_*-*pgdS* using one-step cloning kit, resulting in plasmids pHT-*SP_amyQ_*-*pgdS* and pHT-*SP_aprE_*-*pgdS*.

All recombinant plasmids were verified by DNA sequencing (Sangon Co., Ltd., China). Then, the verified plasmids were transformed into *B. subtilis* 168 as previously described (Young and Spizizen, 1963). Mutant strains were randomly selected from LB agar plates and verified by PCR. All plasmids and strains used in this work are listed in Supplementary Table S2.

### 2.3 Assay of activity of PgdS

The activity of PgdS was measured as previously described (Sha et al., 2018). Briefly, crude enzyme solution was collected from culture by centrifugation. The 0.5 mL of reaction mixture contained 50 mM phosphate buffer (pH 7.0), 1 g/L γ-PGA and 100 μL crude enzyme solution, and was incubated at 37 °C for 2 h. The reaction was terminated by boiling for 5 min. Then 1 mL of 2 M acetate buffer and 1 mL ninhydrin chromogenic solution were added into the reaction, and was incubated at 100 °C for 15 min. The UV absorption of reaction was measured at 570 nm. The amount of free amino groups was calculated. One unit of PgdS was defined as the amount of enzyme that generated 1 μM of free amino groups per hour.

### 2.4 Purification and analysis of γ-PGA

The culture was centrifuged at 12,000 g for 30 min to remove the bacteria, the supernatant was mixed with 4 times the volume of 95% ethanol, the precipitate was collected by centrifugation at 12,000 g for 5 min and redissolved with deionized water. Then, the solution was centrifuged at 12,000 g for 5 min to remove insoluble impurities, the supernatant was transferred into dialysis bag (aperture: 10 kDa) overnight. Finally, γ-PGA was obtained through lyophilization.

For Mw of γ-PGA analysis, the purified γ-PGA was prepared in 0.3 M Na_2_SO_4_ as a 1.0 mg/mL aqueous solution. The Mw of γ-PGA was analyzed using gel permeation chromatography (GPC), equipped with a TSKgel G6000 PWXL column (TosohBioscience, Japan). The mobile phase consisted of 0.3 M Na_2_SO_4_ solution in water, with a flow rate of 0.5 mL/min at 30 °C, and detection was performed with a refractive index detector.

For stereochemical γ-PGA analysis, the purified γ-PGA was dissolved in 6 M HCl and incubated at 105 °C for 12 h. Then, the solution pH was adjusted to 7.0 by 6 M NaOH. Samples was analyzed by HPLC (Shimadzu, Japan), equipped with Chialpak MA(+) (Daicel Corp, Japan). The mobile phase consisted of 2 mM CuSO_4_ solution in water, with a flow rate of 0.5 mL/min at 25 °C, and detection at 254 nm.

For NMR analysis, the 10 mg of purified γ-PGA was dissolved in 500 μL of D_2_O. Spectra were recorded using NMR spectrometer (Avance III HD600, Bruker).

The γ-PGA titer of the fermentation broth was analyzed as previously described (Zeng et al., 2012). Briefly, the cell was removed by centrifugation (12,000 g), and the supernatants were collected. Then, four volumes of ethanol were added in supernatants. The mixture was centrifuged at 12,000 g to remove supernatants and collect γ-PGA-containing precipitate. Next, the precipitate was dried and redissolved in deionized water. Finally, the UV absorption of γ-PGA-containing aqueous solution was detected at 216 nm. The γ-PGA titer was calculated by the standard curve.

### 2.5 Statistical analysis

Statistical significance was performed using analysis of ANOVA with a significance level of 0.05 by SPSSAU (www.spssau.com).

## 3 Results

### 3.1 Construction of engineered strains for γ-PGA production

**Figure 1:**
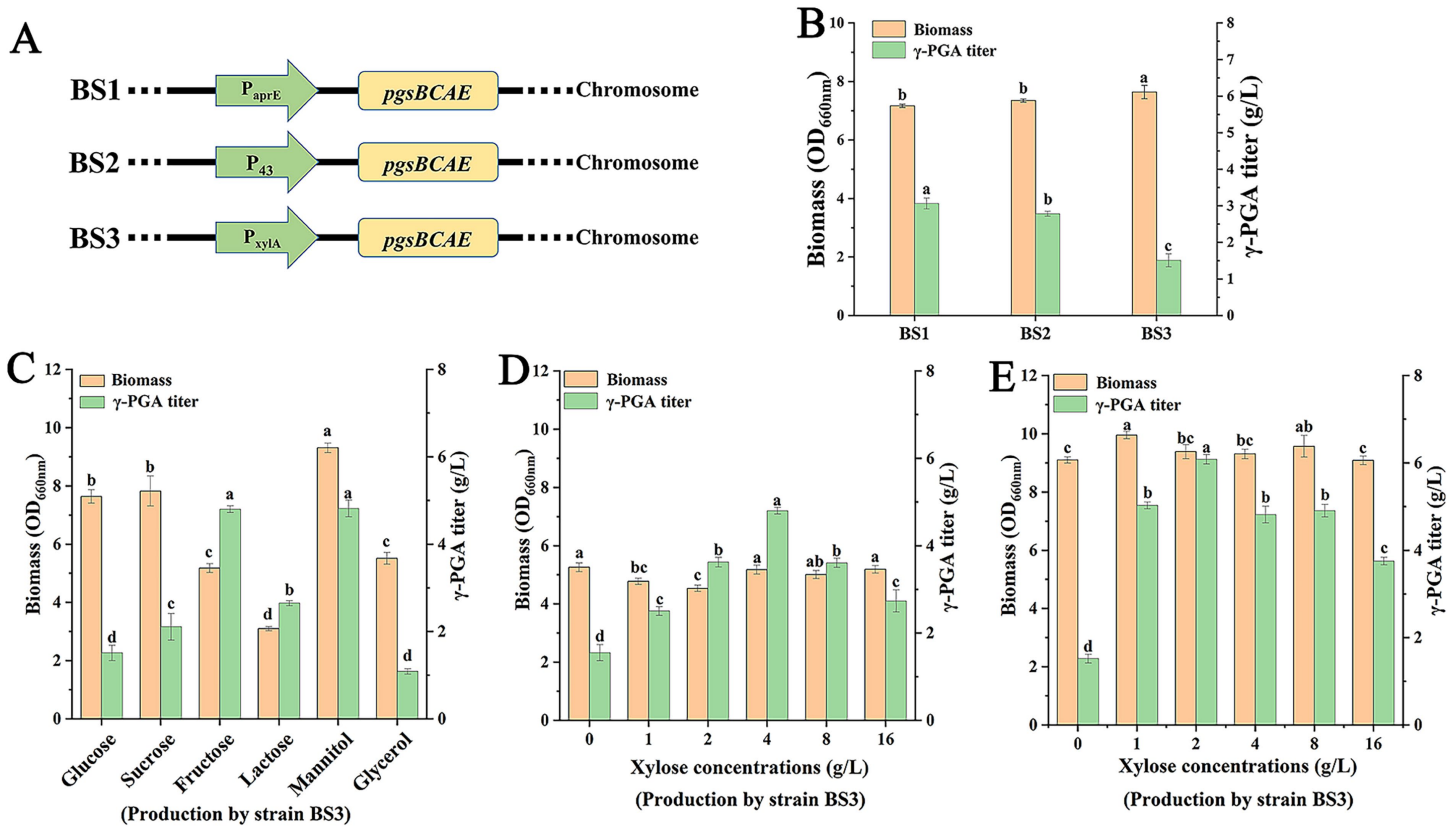
Production of the γ-PGA by engineered strains. **(A)** Schematic diagram of integration expression by different promoters. **(B)** Production of γ-PGA by strains BS1, BS2 and BS3. For strain BS3, 4 g/L xylose was added to the cultures at 8 h. **(C)** Effect of carbon source on γ-PGA production by strain BS3. Xylose (4 g/L) was added to the cultures at 8 h. **(D)** Effect of xylose concentrations on γ-PGA production by strain BS3 when fructose was used as carbon source. **(E)** Effect of xylose concentrations on γ-PGA production by strain BS3 when mannitol was used as carbon source. Different letters (a–d) are annotated on graphs to indicate statistical significance among treatments at *p* < 0.05.

Although the genome of *B. subtilis* 168 contains the γ-PGA biosynthesis gene cluster *pgsBCAE*, it is usually not transcribed (Urushibata et al., 2002). Therefore, the constitutive promoters P_aprE_ and P_43_ and inducible promoter P_xylA_ were integrated chromosome of the *B. subtilis* 168 to produce strains BS1, BS2, and BS3 (Figure 1A), respectively. Next, the engineered strains were used for γ-PGA production. The results showed that titers of γ-PGA in BS1, BS2, and BS3 were 3.06 g/L, 2.78 g/L, and 1.51 g/L (Figure 1B), respectively, suggesting that promoter P_aprE_ may have stronger transcription ability than P_43_ and P_xylA_.

However, the promoter P_xylA_ can be inhibited by glucose in fermentation (Kim et al., 1996). Therefore, we selected a variety of carbon sources, including sucrose, fructose, lactose, mannitol and glycerol, to replace glucose in fermentation for elimination of glucose inhibition. All the other components of the media remained the same. As shown in Figure 1C, when medium contained sucrose, the biomass and γ-PGA titer were 7.82 and 2.1 g/L, respectively. Compared with glucose, sucrose did not improve the γ-PGA production. The reason was that sucrose was hydrolyzed to produce glucose and fructose in fermentation, producing glucose inhibited the P_xylA_. When fructose and mannitol were carbon sources, the corresponding γ-PGA titers were 4.8 g/L and 4.82 g/L, respectively, which both were higher than those of strains BS1 and BS2. Notably, the OD_660_ was highest at 9.31 in 48 h by using mannitol, suggesting mannitol was more favorable to the cell growth. In addition, both biomass and γ-PGA titer decreased by using glycerol, indicating that glycerol was not suitable to γ-PGA production for strain BS3. Considering that there was no significant difference between fructose and mannitol as carbon sources in γ-PGA production, fructose and mannitol were selected as carbon sources for further research.

Since the P_xylA_ promoter was induced by xylose, the effect of xylose concentration in fermentation was studied when carbon sources were fructose and mannitol, respectively. As shown in Figure 1D, with fructose as carbon source and concentration of xylose was 4 g/L, the γ-PGA titer was maximum at 4.8 g/L. However, with mannitol as carbon source and concentration of xylose was 2 g/L, the γ-PGA titer was 6.08 g/L (Figure 1E). Compared with fructose, mannitol increased γ-PGA production by 26.67% while requiring less xylose. Therefore, mannitol had good performance on γ-PGA production in strain BS3.

Furthermore, the colony morphology of *B. subtilis* 168 and BS3 were observed. The results showed that *B. subtilis* 168 formed big and flat colonies (Supplementary Figure S1A). In contrast, the colonies of BS3 appeared globoid and viscous, indicating that it has the ability to synthesize γ-PGA. Next, the purified γ-PGA was analyzed by NMR (Supplementary Figure S1B). These results determined that purified product in this study was γ-PGA. In addition, stereochemical analysis of γ-PGA showed that D/L-glutamic acid ratio was 59: 41 (Supplementary Figure S1C).

### 3.2 Effect of deletion of γ-PGA hydrolases on γ-PGA production

**Figure 2:**
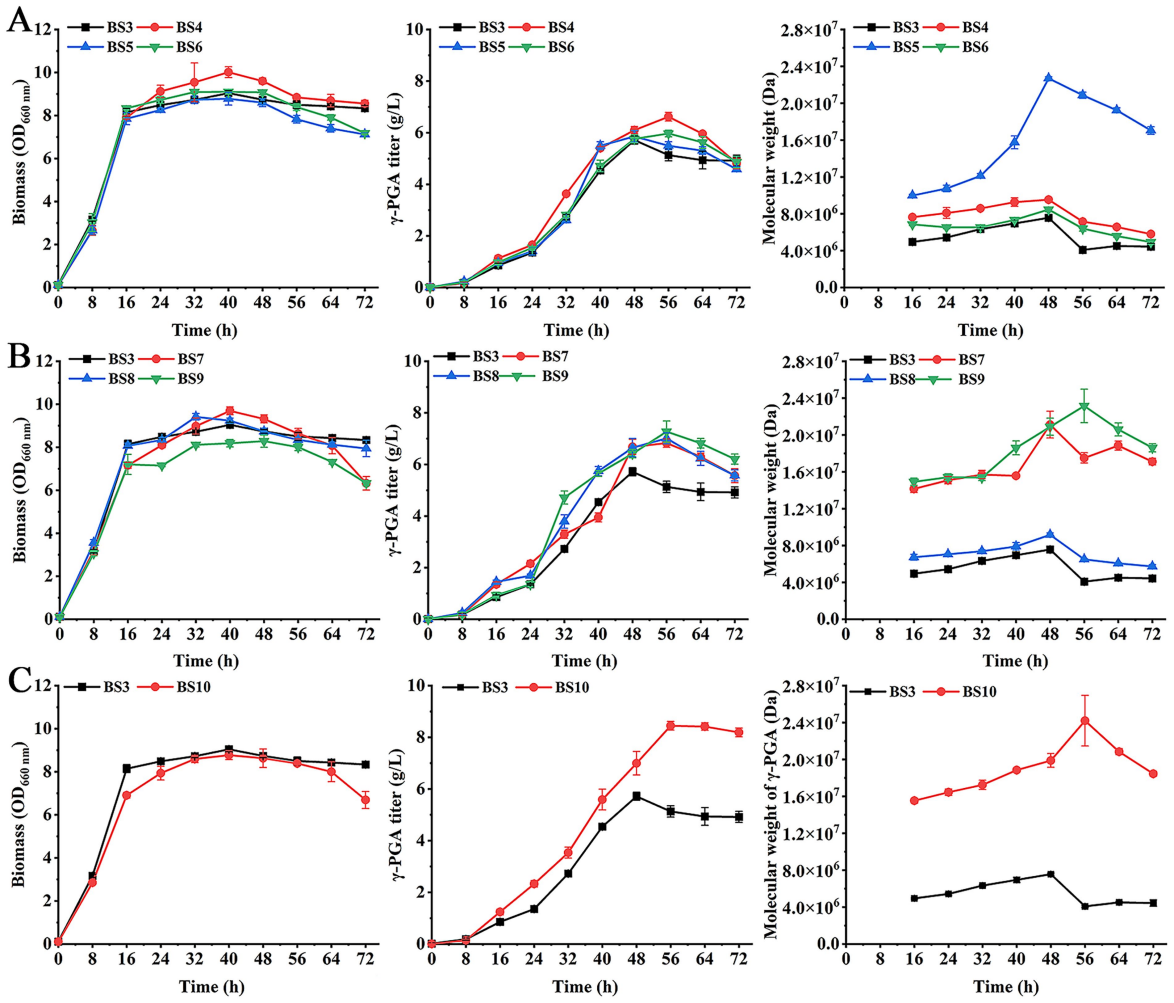
Effect of hydrolase genes deletion on the biomass, γ-PGA titer and Mw. **(A)** Single-gene deletion strains: BS4 (BS3Δ*cwlO*), BS5 (BS3Δ*pgdS*), BS6 (BS3Δ*ggt*). **(B)** Double-gene deletion strains: BS7 (BS3Δ*cwlO*Δ*pgdS*), BS8 (BS3Δ*cwlO*Δ*ggt*) and BS9 (BS3Δ*ggt*Δ*pgdS*). **(C)** Triple-gene deletion strain: BS10 (BS3Δ*cwlO*Δ*ggt*Δ*pgdS*).

The *B. subtilis* contains hydrolases CwlO, PgdS and GGT. These enzymes can degrade γ-PGA to affect the Mw and concentration of γ-PGA (Mitsui et al., 2011; Scoffone et al., 2013). In order to increase the γ-PGA Mw and titer, the effects of deletion of genes *cwlO, pgdS* and *ggt* on γ-PGA production were investigated. Recombinant strains BS4 (BS3Δ*cwlO*), BS5 (BS3Δ*pgdS*), and BS6 (BS3Δ*ggt*) were constructed and used to produce γ-PGA under optimal culture conditions (fermentation medium containing 30 g/L mannitol, 2.5 g/L yeast extract, 20 g/L L-glutamate, 1 g/L K_2_HPO_4_, 1 g/L MgSO_4_, pH 7.0; 2 g/L xylose added to the cultures at 8 h). The results showed that biomass of BS4 was higher than BS3 and other mutant strains (Figure 2A), while BS5 and BS6 showed no significant difference from BS3. The γ-PGA titer in BS4 was highest at 6.62 g/L at 56 h, and its Mw was 9.53 × 10^6^ Da. In addition, γ-PGA Mw of BS5 were highest at 2.27 × 10^7^ Da at 48 h, which increased by 200.26% compared to BS3. These results indicated that deletion of *cwlO* improved the γ-PGA titer, while PgdS had a significant impact on the Mw of γ-PGA. In contrast, deletion of *ggt* did not substantially enhance either the titer or the Mw.

Next, the double-gene deletion of γ-PGA hydrolases was conducted. The strains BS7 (BS3Δ*cwlO*Δ*pgdS*), BS8 (BS3Δ*cwlO*Δ*ggt*) and BS9 (BS3Δ*ggt*Δ*pgdS*) were constructed and used for γ-PGA production. The results showed that there was no significant difference in the biomass between strains BS7, BS8 and BS3 (Figure 2B). The biomass of strain BS9 was slightly lower than that of BS3 and other double-gene deletion strains after 48 h. The maximum titers of γ-PGA in BS7, BS8 and BS9 were 6.83 g/L, 7.01 g/L and 7.27 g/L at 56 h, respectively, which were 19.41, 22.56 and 27.1% higher than that of BS3. The γ-PGA Mw in BS7 and BS9 obviously higher than BS8 and BS3. BS9 achieved the highest Mw at 2.31 × 10^7^ Da at 56 h. Although it was higher than BS7 (2.11 × 10^7^ Da) and BS8 (9.17 × 10^6^ Da), it was only 1.8% higher than single-gene deletion strain BS5 (2.27 × 10^7^ Da). These results suggested that all double-gene deletion strains increased the titer and Mw of the generated γ-PGA compared to BS3.

Furthermore, the three genes *cwlO, pgdS* and *ggt* were knocked out to produce strain BS10 (BS3Δ*cwlO*Δ*ggt*Δ*pgdS*). Production of γ-PGA by strain BS10 was assayed. As shown in Figure 2C, the biomass of BS10 was slightly lower than BS3. But γ-PGA titer and Mw were 8.44 g/L and 2.42 × 10^7^ Da at 56 h, respectively, which increased by 47.81 and 220.1%. These results determined that deletion of *cwlO, pgdS*, and *ggt* can further increase the γ-PGA titer and Mw.

### 3.3 Regulation of PgdS expression level for production of γ-PGA with different Mws

**Figure 3:**
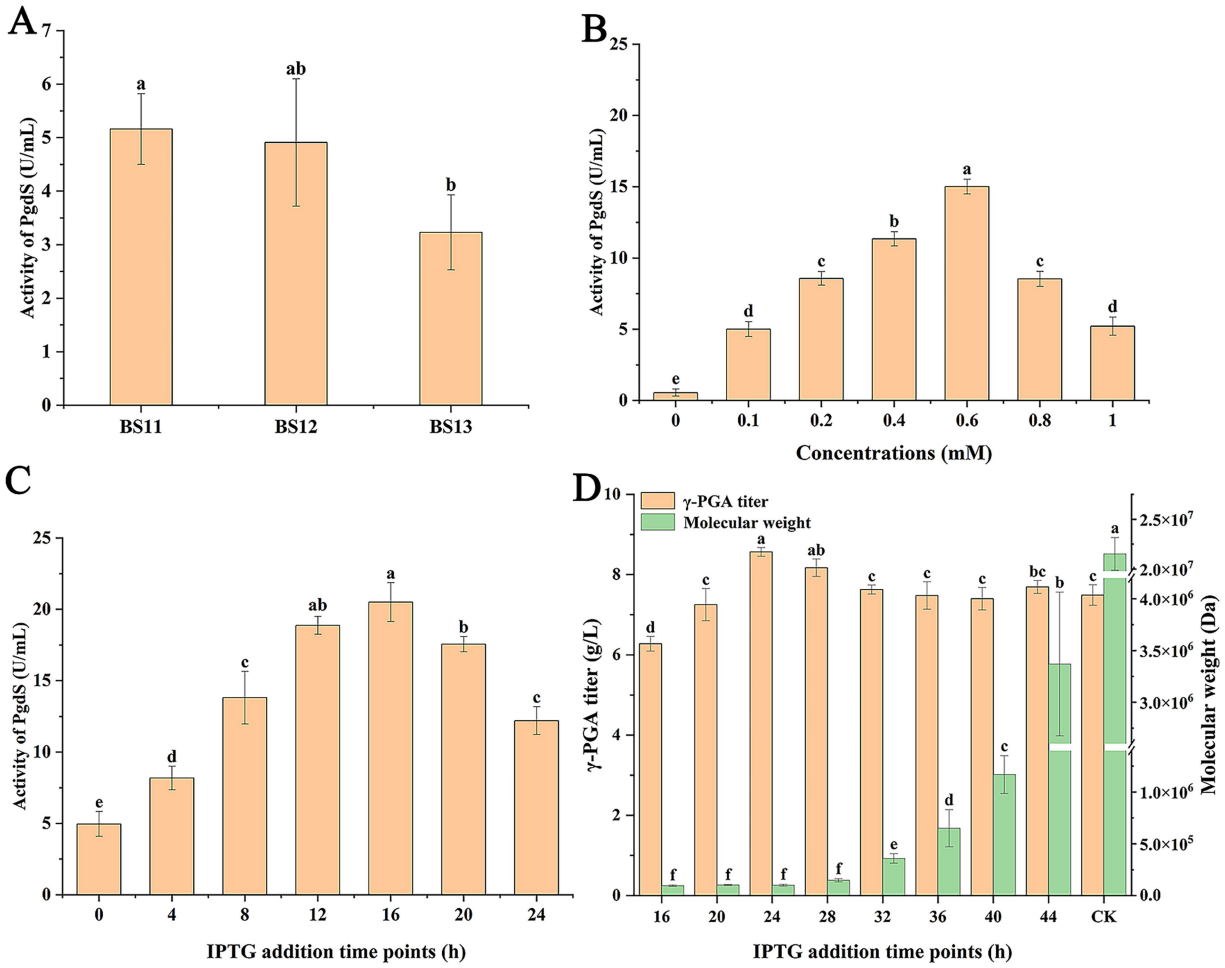
Effect of PgdS activity in culture on γ-PGA production. **(A)** Effect of signal peptide on PgdS activity in culture. **(B)** Effect of IPTG concentrations on PgdS activity in culture. **(C)** Effect of IPTG addition time points on PgdS activity in culture. **(D)** Effect of IPTG addition time points on γ-PGA production. CK: no IPTG addition. Different letters (a–f) are annotated on graphs to indicate statistical significance among treatments at *p* < 0.05.

In our present study, it has been determined that PgdS had a significant effect on the γ-PGA Mw. So we attempted to regulate expression level of PgdS for control of the Mw of generated γ-PGA. The inducible promoter P_grac_ by IPTG was selected to control PgdS expression level. The plasmid pHT-*SP_PgdS_*-*pgdS* was constructed and transformed into BS10 to produce strain BS11. In addition, PgdS contains a signal peptide SP_PgdS_ (amino acids codes: 1–32) that transport PgdS into the culture to hydrolyze γ-PGA. Considering that the efficiency of PgdS secretion affects its activity in the culture, we sought to enhance PgdS secretion to produce γ-PGA with lower Mw, thereby expanding the range of γ-PGA Mw. Therefore, the signal peptides SP_amyQ_ and SP_aprE_ replaced SP_PgdS_ to construct strains BS12 and BS13, respectively. The specific activity of PgdS in supernatant of culture was measured. The results showed that the specific activities of PgdS in strains BS11, BS12, and BS13 were 5.16 U/mL, 4.91 U/mL, and 3.23 U/mL (Figure 3A), respectively, suggesting that SP_PgdS_ was more favorable for PgdS secretion. Thus strain BS11 was selected for further study.

Next, the effect of IPTG concentrations on PgdS activity were studied. As shown in Figure 3B, when concentrations of IPTG were 0–0.6 mM, the activity of PgdS increased with increasing IPTG concentrations. However, when IPTG concentrations exceeded 0.6 mM, the activity of PgdS decreased. Therefore, 0.6 mM IPTG was used as an optimal concentration. In optimal IPTG concentration, effect of IPTG addition time on PgdS activity was assayed. The results showed that PgdS activity in supernatant was highest at 20.51 U/mL when IPTG was added at 16 h (Figure 3C).

Finally, the effect of IPTG addition time on γ-PGA production was studied. The results showed that the Mw of generated γ-PGA was related to IPTG addition time (Figure 3D). When IPTG was not added, the γ-PGA titer and Mw in BS11 were 7.49 g/L and 2.15 × 10^7^ Da, respectively. Compared with control, the γ-PGA Mw decreased to 9.55 × 10^4^ Da, 9.95 × 10^4^ Da, and 6.5 × 10^5^ Da, respectively, when IPTG was added at 16 h, 24 h, and 36 h. However, the γ-PGA titer increased with IPTG addition time increase (16–24 h). The γ-PGA titer was lowest at 6.28 g/L when IPTG was added at 16 h. When IPTG was added after 32 h, γ-PGA titer was stable. These results suggested that change of IPTG addition time can produce γ-PGA with specific Mw in an engineered strain. In summary, the γ-PGA with Mw ranging from 9.55 × 10^4^ Da to 2.15 × 10^7^ Da was produced by an engineered strain, with the titer of 6.28–8.57 g/L.

### 3.4 Production of γ-PGA in 5-L fermenter

To explore the production potential of strain BS11, the 5-L fermenter was used for large-scale experiments. The results showed that the γ-PGA Mw and titer were 1.85 × 10^7^ Da and 4.86 g/L at 56 h (Supplementary Figure S2), respectively, which were lower than shake flask. Considering the many factors have an impact on fermentation, such as inoculation amount, temperature, aeration rate, stirring speed and pH. Therefore, the fermentation conditions of γ-PGA should be optimized.

**Figure 4:**
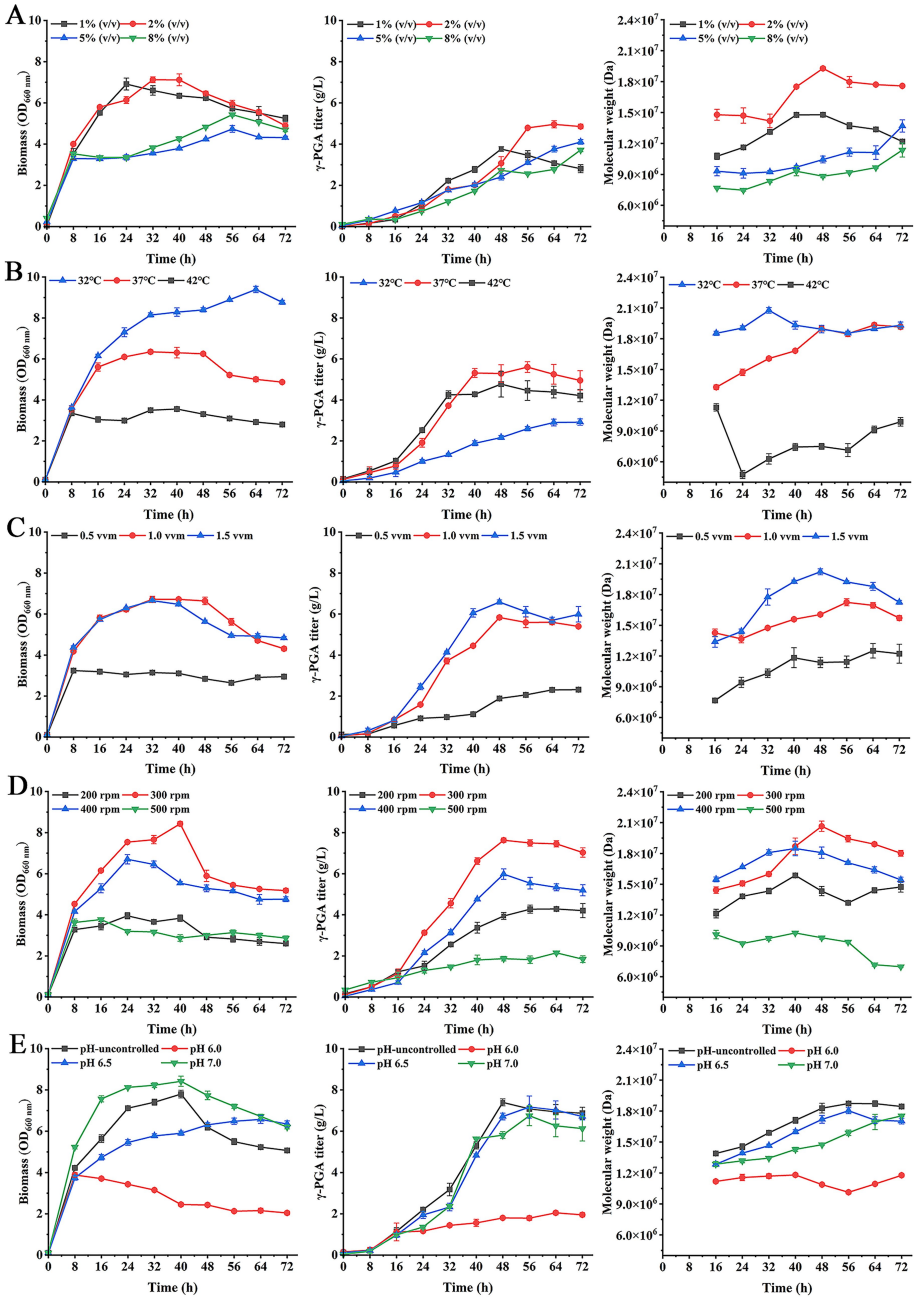
Effects of inoculation amounts **(A)**, temperature **(B)**, aeration rate **(C)**, agitation speed **(D)** and pH **(E)** on γ-PGA production in 5-L fermenter.

First, the inoculation amounts were set 1% (v/v), 2, 5, and 8% in fermentation. As shown in Figure 4A, when inoculation amounts were 1 and 2%, respectively, their biomass was higher than the 5 and 8% of inoculation amounts. The γ-PGA titer was highest at 4.96 g/L in fermentation with 2% of inoculation amount. The γ-PGA Mw in 2% of inoculation amount was highest at 1.93 × 10^7^ Da. These results suggested that when inoculation amount was 2%, the efficiency of γ-PGA synthesis was highest.

Then, in optimal inoculation amount, the effect of temperature on γ-PGA production was studied. The results showed that when temperature was 32 °C, the biomass was higher than at 37 °C and 42 °C (Figure 4B). However, the γ-PGA titer was lowest at 32 °C. The γ-PGA titer kept stable after 40 h at 37 °C, its maximum titer was 5.6 g/L. In addition, the γ-PGA Mw at 32 °C was higher than at 37 °C and 42 °C during 0–40 h. But the γ-PGA Mw at 37 °C increased with the increase of fermentation time and showed no significant difference from that at 32 °C after 48 h. Considering γ-PGA Mw and titer, the 37 °C temperature was selected for optimal temperature.

In the optimal inoculation amount and temperature, the effect of aeration rate on γ-PGA production was explored. As shown in Figure 4C, the cell growth was faster at aeration rate of 1.0 vvm and 1.5 vvm, corresponding to higher titers than 0.5 vvm. The γ-PGA titer was highest at 6.58 g/L at 1.5 vvm. The γ-PGA Mw at 1.5 vvm significantly higher than that of 0.5 vvm and 1.0 vvm, with a highest Mw of 2.02 × 10^7^ Da. So the 1.5 vvm was used for optimal aeration rate.

In optimal inoculation amount, temperature and aeration rate, the agitation speed in fermentation was optimized. The results showed that the cell growth was better at 300 rpm and γ-PGA titer was maximum at 7.63 g/L compared to other agitation speeds (Figure 4D). The γ-PGA Mw was highest at 2.06 × 10^7^ Da at 300 rpm. These results indicated that 300 rpm was more conducive to γ-PGA production.

Based on above optimal conditions, the effect of pH on γ-PGA production was investigated. As shown in Figure 4E, although biomass at pH 7.0 was higher, the γ-PGA titer was maximum at 7.4 g/L when pH was not controlled. The γ-PGA Mw was highest at 1.87 × 10^7^ Da. Therefore, the production efficiency of γ-PGA was better in the pH-uncontrolled group, which also reduced the cost.

**Figure 5:**
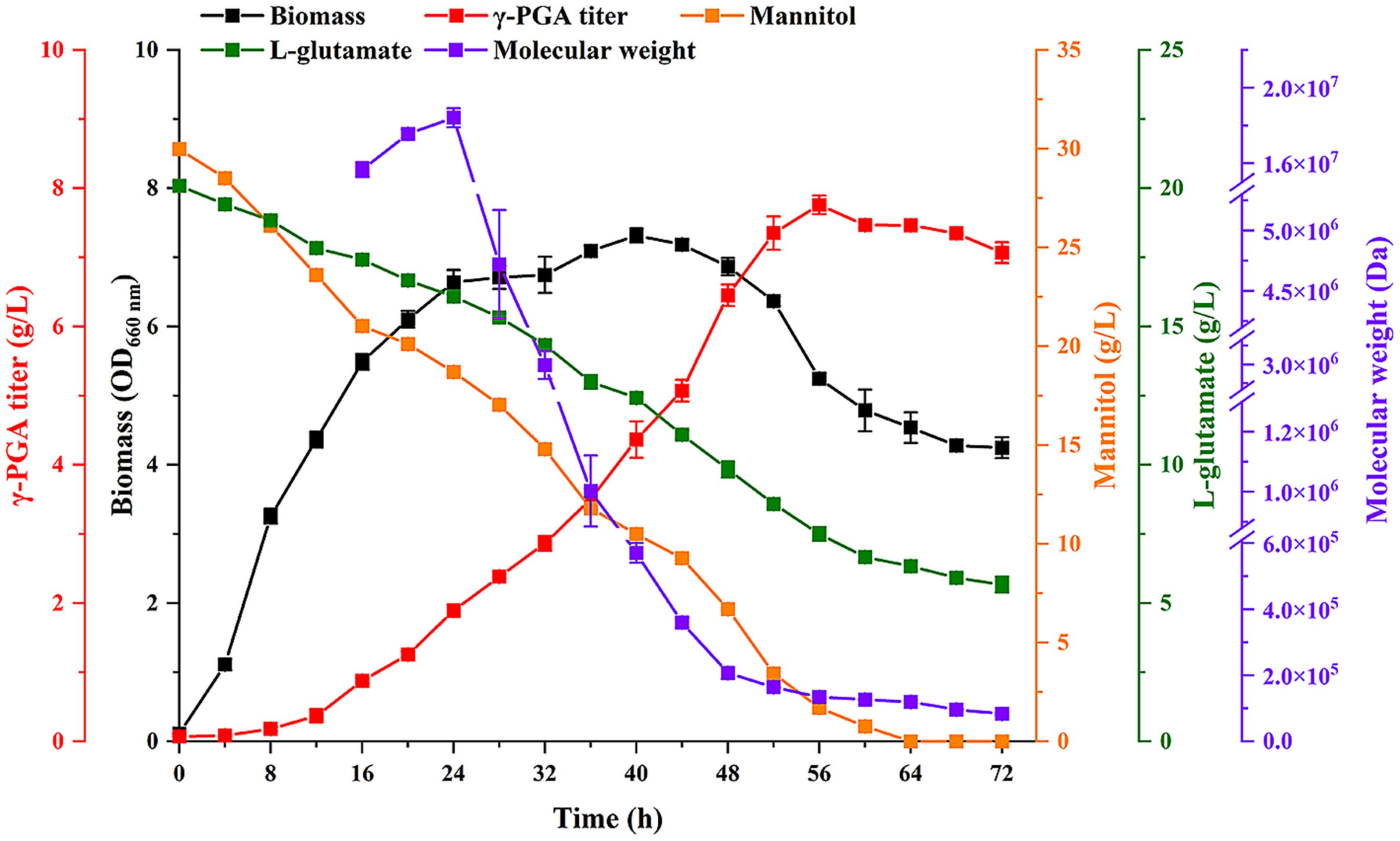
Production of γ-PGA in 5-L fermenter at optimal culture conditions.

Finally, the optimal culture conditions were further applied to produce γ-PGA in 5-L fermenter. Meanwhile, IPTG was added at 0.6 mM in culture at 24 h. The results showed that the biomass at 40 h was the highest (Figure 5), and then decreased. The maximum titer was 7.76 g/L at 56 h. The γ-PGA Mw increased from 8 to 24 h, with a highest Mw was 1.84 × 10^7^ Da. Then γ-PGA Mw decreased after IPTG addition. The lowest Mw was 8.3 × 10^4^ Da at 72 h. These results suggested that IPTG-induced expression of PgdS to change γ-PGA Mw was feasible in large-scale experiments. Although IPTG was only added at 24 h, we are confident that γ-PGA with specific Mw can be produced by changing the IPTG addition time in large-scale experiments in future study.

## 4 Discussion

In this study, we activated the expression of the γ-PGA synthetase gene cluster of *B. subtilis* 168 and expanded the Mw range of the produced γ-PGA by deleting hydrolases and overexpressing PgdS.

Previous studies have demonstrated that glucose competed with xylose for inhibition of xylose-binding sites on the promoter P_xylA_ in *B. subtilis*, thereby inhibiting transcription (Kim et al., 1996). In addition, γ-PGA synthesis also requires hydrolysis of ATP to provide energy for the reaction (Qiu et al., 2023). Thus, many studies used cheap glucose as carbon source for γ-PGA biosynthesis (Halmschlag et al., 2020; Xu et al., 2024). However, in this study, glucose may not be a suitable carbon source for strains utilizing the P_xylA_ promoter to regulate γ-PGA synthetases expression. Therefore, screening for a more suitable carbon source to support both cell growth and γ-PGA production was necessary. We found that mannitol, as a carbon source, used less xylose and resulted in a higher γ-PGA titer compared to other carbon sources. Specifically, γ-PGA titer increased by about three times relative to the initial culture conditions. This improvement may be attributed to the elimination of glucose-mediated inhibition. Previous research also reported that mannitol served as the optimal carbon source for γ-PGA production in *B. methylotrophicus*, likely by affecting biosynthesis of endogenous glutamic acid through the TCA cycle (Chatterjee et al., 2017). We speculated that a similar mechanism may occur in *B. subtilis*.

Next, we found that strain BS3 produced ultra-high-Mw γ-PGA (7.56 × 10^6^ Da). In previous reports, γ-PGA with a Mw of 8.5 × 10^6^ Da was also produced by using *B. subtilis* 168 as a host (Halmschlag et al., 2019). However, this ultra-high-Mw γ-PGA has rarely been produced in other *Bacillus* strains, such as *B. licheniformis* WX-02 and *B. amyloliquefaciens* NB, which only produce γ-PGA with Mw values of 1.2 × 10^6^ Da and 1.38 × 10^6^ Da, respectively (Tian et al., 2014; Sha et al., 2018). These findings suggest that the production of ultra-high-Mw γ-PGA in *B. subtilis* 168 may be more suitable than in other strains.

To overcome the limitations of the strain on the maximum Mw, we investigated the effects of deletion of hydrolases CwlO, PgdS, and GGT genes in *B. subtilis* on γ-PGA production. Previous studies have shown that the deletion of *cwlO*, *pgdS*, and *ggt* did not necessarily increase the Mw or titer of γ-PGA in the other *Bacillus* strains (Feng et al., 2014; Ojima et al., 2019). Therefore, we performed the single, double and triple deletions of these genes. The results showed that both Δ*pgdS* and Δ*ggt* did not significantly improve the titer of γ-PGA. Only Δ*cwlO* increased the titer of γ-PGA by 15.93%. A similar trend (27.64% increase) was reported in *B. amyloliquefaciens* LL3 following *cwlO* deletion (Feng et al., 2014). This may be that due to the higher biomass of the Δ*cwlO* strain. Furthermore, the deletion of *cwlO* may lead to the cells shorter than wild-type strain, resulting in a higher specific surface area, which would increase the γ-PGA transport, therefore improving its γ-PGA production. In addition, since γ-PGA is formed by an enzyme complex in the cell membrane, a larger membrane surface area may lead to more γ-PGA formation (Feng et al., 2014; Hoffmann et al., 2022). GGT was expressed during the stationary phase of cell and hydrolyzed γ-PGA to produce D/L-glutamic acid monomer, which provided nutrients for cells (Kimura et al., 2004; Scoffone et al., 2013). Therefore, the short hydrolysis time and exo-hydrolase activity of GGT may indicate no significant improvement in the titer and Mw of γ-PGA in the Δ*ggt* strain. Similarly, the deletion of *ggt* had minimal effect on γ-PGA titer in *B. amyloliquefaciens* LL3 (Feng et al., 2014). In contrast, Δ*ggt* improved γ-PGA production in *B. licheniformis* RK14-46, while Δ*pgdS* inhibited it (Ojima et al., 2019). In our study, the deletion of *pgdS* significantly increased the γ-PGA Mw by 200.26%, whereas deletions of *cwlO* and *ggt* had no significant effect. These results indicated that PgdS played an important role in regulating the Mw of γ-PGA. Notably, this an extent of Mw increase was not observed in *B. amyloliquefaciens* and *B. licheniformis* (Feng et al., 2014; Sha et al., 2020b; Wang et al., 2020). Moreover, we found that the triple-deletion strain BS10 exhibited a marked improvement in both γ-PGA titer and Mw, with increases of 47.81 and 220.1%, respectively. In contrast, the triple deletions of *cwlO*, *ggt*, and *pgdS* in *B. amyloliquefaciens* LL3 resulted in a 27.1% decrease in γ-PGA titer and only a 16.77% increase in Mw (Feng et al., 2014). Similarly, in *B. amyloliquefaciens* NBCSO-3 (Δ*cwlO*Δ*ggt*Δ*pgdS*), the γ-PGA titer declined, while the γ-PGA Mw showed only a slight increase (Sha et al., 2020b). These findings suggest that *B. subtilis* 168 is a promising chassis for expanding the regulation range of γ-PGA Mws.

Regulating the expression level of PgdS has been a typically strategy for controlling the Mw of γ-PGA. Previous studies have used constitutive promoters or phase-dependent promoters to control PgdS expression level. However, these methods lacked efficient dynamic regulation of PgdS expression level, often requiring the construction of multiple engineered strains for γ-PGA with variable Mws (Wang et al., 2020; Chen et al., 2023). In addition, some reports focused on the production of low-Mw γ-PGA by enhancing PgdS expression (Tian et al., 2014; Sha et al., 2018; Sha et al., 2020a). In this study, we constructed strain BS11 for PgdS expression under the control of an IPTG-inducible promoter P_grac_. The results demonstrate that the γ-PGA Mw can be dynamically regulated with change of IPTG addition time. Although P_grac_ has been previously used in *B. amyloliquefaciens* NBCSO-3 (Δ*pgdS*Δ*cwlO*) to regulate PgdS expression, it was reported that the exchange of the additional time of IPTG had a little effect on PgdS activity. Therefore, a CRISPRi system was further introduced in *B. amyloliquefaciens* NBCSO-3 (Δ*pgdS*Δ*cwlO*) to construct an engineered strain that can produce γ-PGA with variable Mw (Sha et al., 2020b). In that system, dCas was regulated by an IPTG-inducible promoter, while three sgRNAs were controlled by xylose-, maltose-, and arabinose-inducible promoters, respectively. As a result, the production of γ-PGA with specific Mws required adjusting the concentrations of multiple inducers in the culture, making the operation relatively complicated. In contrast, our study provides a simple and effective method for production of γ-PGA with wider range of Mw regulation (Table 1). However, the production of γ-PGA with lower Mw in this study was not better than other reports. We speculate that the activity of PgdS from *B. subtilis* 168 is too low to efficiently hydrolyze γ-PGA. Thus, screening the PgdS with high activity may achieve the production of γ-PGA with lower Mw in the future.

**Table 1:** Comparison of the Mw of γ-PGA produced by engineered strains constructed with different strategies.

Microbial hosts	Processes	Titer (g/L)	Mw (Da)	References
*B. subtilis* 168	Dynamic regulation of PgdS expression level in an engineered strain	6.28–8.57	9.55 × 10^4^–2.15 × 10^7^	This study
*B. subtilis* 168	Expressing different γ-PGA synthetases in strains PG27, PG25-E and PG10, respectively	0.2–2	2.9 × 10^4^–3.4 × 10^4^, 1.7 × 10^5^–6.6 × 10^5^, Up to 8.5 × 10^6^	Halmschlag et al. (2019)
*B. subtilis* KH2	Regulation of PgdS expression level in four engineered strains KH2-RA, KH2-RC, KH2-RX, and KH2-RS	13.57–23.28	4.12 × 10^5^, 1.36 × 10^6^, 2.23 × 10^6^, 2.41 × 10^6^	Chen et al. (2023)
*B. licheniformis* WX-02	Construction of multiple strains for regulation of expression and secretion of PgdS	27–34	6.82 × 10^4^–1.78 × 10^6^^a^	Wang et al. (2020)
*B. amyloliquefaciens* NB	Overexpressing PgdS in an engineered strain	17.62	2 × 10^4^–3 × 10^4^	Sha et al. (2018)
*B. amyloliquefaciens* NS	CRISRi system controlling PgdS expression level in an engineered strain	25–27	5 × 10^4^–1.4 × 10^6^	Sha et al. (2020b)

a The lowest and highest Mw of γ-PGA were 6.82 × 10^4^ and 1.78 × 10^6^ Da, respectively, which were produced by strains SP18 and SP43. The data of others strains were not shown.

In addition, we found that γ-PGA titer increased with the duration of IPTG addition (16–24 h). This may be a prolonged expression of PgdS that may compete for the energy and substrate required for the γ-PGA synthesis. However, the γ-PGA titer was highest when IPTG added at 24 h, and then γ-PGA titer decreased with the increase of IPTG addition time. At this point, the broth became very viscous with the Mw of γ-PGA increased gradually, which hindered the oxygen supply and subsequently reduced the supply of ATP for γ-PGA synthesis (Regestein née Meissner et al., 2017; Sirisansaneeyakul et al., 2017). Thus, the production of γ-PGA may be improved by mechanical agitation in the bioreactor.

Finally, we investigated the productivity of strain BS11 in the large-scale production. The maximum γ-PGA titer in 5-L fermenter was 4.86 g/L, which was lower than that obtained in the shake flask, indicating the need for process optimization. After optimizing the culture conditions, the maximum titer of γ-PGA increased by 59.67% at 7.76 g/L in optimal culture conditions. The generated γ-PGA with Mw ranged from 8.3 × 10^4^ Da to 1.87 × 10^7^ Da, which was approximately the consistent with that on the shake flask. These results demonstrate that production of γ-PGA with wide range of Mw regulation in this study has industrial production potential.

## 5 Conclusion

In summary, we presented a significant advancement in production of γ-PGAs with specific Mws by an engineered strain. The Mw and titer of the generated γ-PGA increased by 220.1 and 47.81% by deletion of hydrolase genes *cwlO*, *pgdS*, and *ggt*, respectively. Furthermore, an engineered strain was constructed to produce γ-PGA with Mw ranging from 9.55 × 10^4^ Da to 2.15 × 10^7^ Da by dynamic regulation of PgdS expression level, with the titer of 6.28–8.57 g/L. Finally, γ-PGA with Mw ranging from 8.3 × 10^4^ Da to 1.87 × 10^7^ Da was produced at optimal conditions in the 5-L fermenter, suggesting that it has industrial application potential.

## Data Availability

The original contributions presented in the study are included in the article/Supplementary material, further inquiries can be directed to the corresponding author.
