# Supplementary material for: Construction of an engineered Bacillus subtilis for production of poly-γ-glutamic acids with specific molecular weights
Source: Front Microbiol. 2025 Sep 3;16:1597704. doi: 10.3389/fmicb.2025.1597704 (PMC12442558; doi:10.3389/fmicb.2025.1597704)
Supplement: Supplementary file 1 [file Data_Sheet_1.docx]

Supplementary Material

#
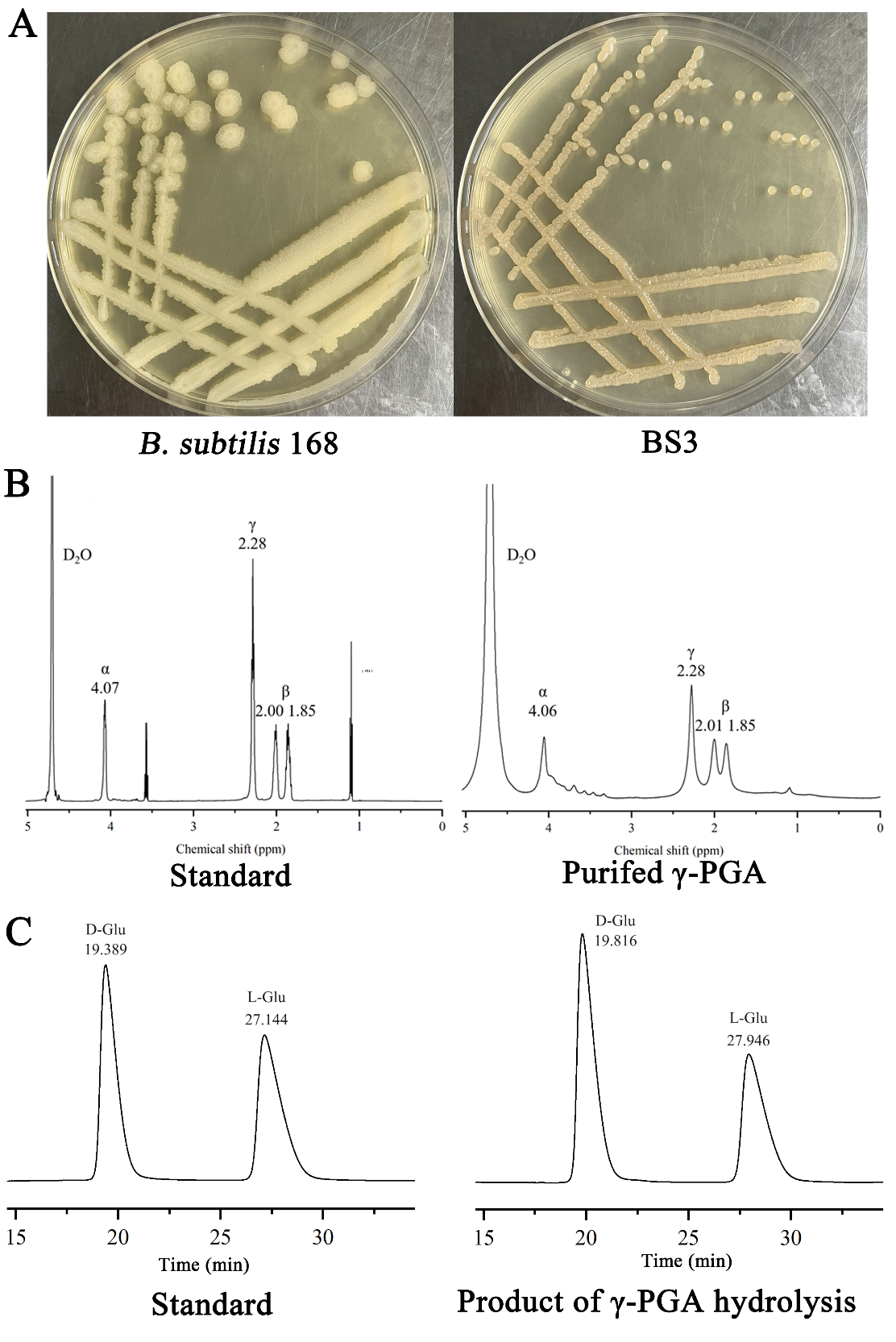


**Supplementary Figure S1.** (A) Comparison of *B. subtilis* 168 and BS3 colony shapes. (B) Analysis of purified γ-PGA by NMR. (C) Analysis of stereochemical structure of γ-PGA by HPLC.

**
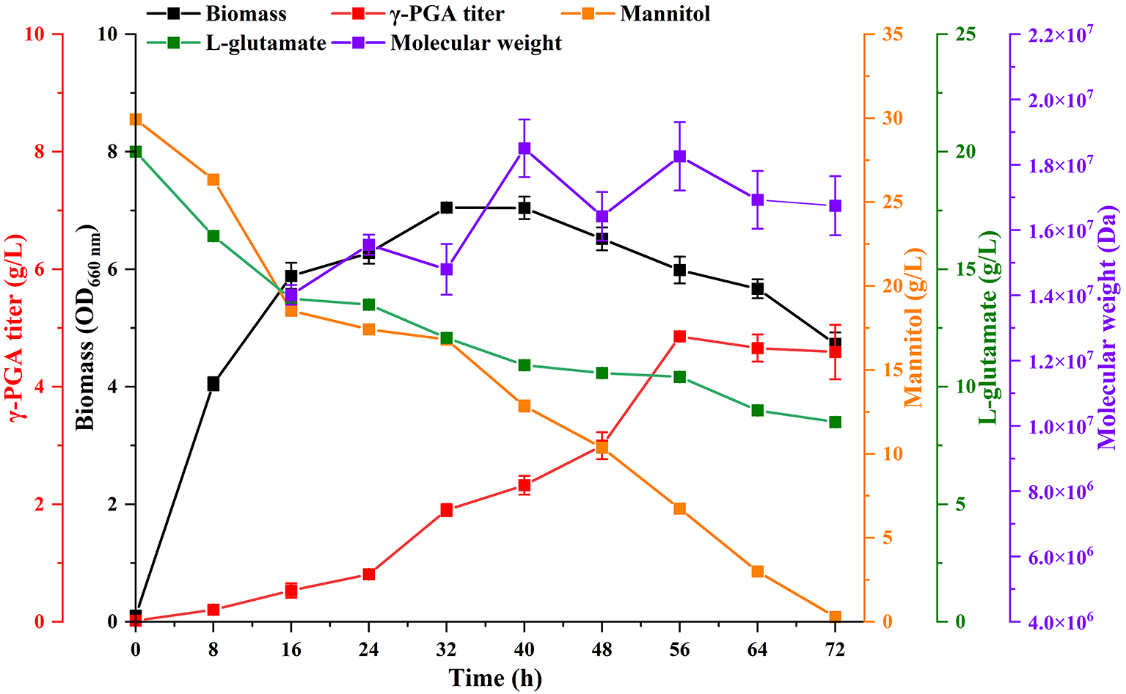
**

**Supplementary Figure S2.** Production of γ-PGA in 5-L fermenter before optimization of culture conditions.

Table S1 Primers used in this study

| Primers | Sequence (5′-3′) |
| --- | --- |
| P43-1 | TTTTAAGCCGTCTGTACGTTCCTAAGAGCTCAGCATTATTGAGTGGATGATTAT |
| P43-2 | AATGAGTAACCACATGTGTACATTCCTCTCTTACCTATAATGGT |
| PxylA-1 | TGAGTAACCACATGTGATTTCCCCCTTAAAAATAAATTCATTCAAATAC |
| PxylA-2 | GGAGATGTCGAAAAGCATTACATTGTAATCATGTCCAGAAAATGATC |
| PaprE-1 | TCTGTACGTTCCTAATTACATTGTAATCATGTCCAGAAAATGA |
| PaprE-2 | AATGAGTAACCACATGTGATTTCCCCCTTAAAAATAAATTCAT |
| U1 | CGACGGCCAGTGCCAAGCTTATCATTACTCGCGATCACACCATCT |
| U2 | TGCTTTTCGACATCTCCTTCTATATTGTTGT |
| D1 | ATGTGGTTACTCATTATAGCCTGTGCTGTCATAC |
| D2 | ACGAATTCGAGCTCGGTACCTTTCAAATGGTACAATCGGGTATAGAAAAT  CAAAC |
| CwlO-U1 | CCAAGCTTGCATGCCTGCAGAAACGGTCAAACTATTAGAGCAAGCTG |
| CwlO-U2 | CAACTAACGTATCCGGAAGCCAAACCAAGTGTAATTAAACT |
| CwlO-D1 | GGTTTGGCTTCCGGATACGTTAGTTGGCAGAGGACAAG |
| CwlO-D1 | TCTTCGTGGTTTTTCAATCTAGATGCCGTCAATGACCTCTTTAATATCG |
| PgdS-U1 | CCAAGCTTGCATGCCTGCAGTTTTCTACAGCCTCGGCAACTTTG |
| PgdS-U2 | GTTCCGGCATTGCTTCCAGTTTGCCAGTGTGTTCAC |
| PgdS-D1 | CAAACTGGAAGCAATGCCGGAACTAAGATTGAGAAGG |
| PgdS-D1 | ATCTTCGTGGTTTTTCAATCTAGAACGGCGGTGAAAACATTTATAAGCT |
| GGT-U1 | CCAAGCTTGCATGCCTGCAGTAGACTGCGAATCGGCTGTACAATATG |
| GGT-U2 | GTAACGGTAAGAGCTTTAAACAGACGTTCCACGTTCTTTTCATG |
| GGT-D1 | GGAACGTCTGTTTAAAGCTCTTACCGTTACGAAGACG |
| GGT-D2 | CTTCGTGGTTTTTCAATCTAGACAGCTGATACGCATCATTTGATTCAAC |
| AmyQ-1 | AAGGAGGAAGGATCAATGATTCAAAAACGAAAGCGGACAG |
| AmyQ-2 | CAGCTTCCGCAATCTCGGCTGATGTTTTTGTAATCGGCAAAC |
| AprE-1 | AAGGAGGAAGGATCAGTGAGAAGCAAAAAATTGTGGATCAG |
| AprE-2 | CAGCTTCCGCAATCTCGCAGCCTGCGCAGACATG |
| PgdS-1 | AAGGAGGAAGGATCAATGAACACACTGGCAAACTGGAAG |
| PgdS-2 | CAGCTTCCGCAATCTCGCTTTTGTCATAATTGGAACCAAA |

Table S2 The plasmids and strains used in this study.

| Plasmids or strains | Description | Ref |
| --- | --- | --- |
| pKSV7 | *E. coli*-*B. subtilis shuttle* vector, Amp^R^, Cmr^R^ | Lab stock |
| pHT43 | *E. coli*-*B. subtilis* expression vector, Amp^R^, Cmr^R^ |  |
| pKSV-*P_43_* | pKSV7 derivate, promoter P_43_, regulating the expression of *pgsBCAE* gene cluster. | This study |
| pKSV-*P_aprE_* | pKSV7 derivate, promoter P_apreE_, regulating the expression of *pgsBCAE* gene cluster. |  |
| pKSV-*P_xylA_* | pKSV7 derivate, promoter P_xylA_, regulating the expression of *pgsBCAE* gene cluster. |  |
| pKSV-*cwlO* | pKSV7 derivate, carrying a mutant copy of the *cwlO* used for deletion of the *cwlO* |  |
| pKSV-*pgdS* | pKSV7 derivate, carrying a mutant copy of the *pgdS* used for deletion of the *pgdS* |  |
| pKSV-*ggt* | pKSV7 derivate, carrying a mutant copy of the *ggt* used for deletion of the *ggt* |  |
| pHT-*SP_PgdS_*-*pgdS* | pHT43 derivate, P_grac_-SP_PgdS_-PgdS |  |
| pHT-*SP_amyQ_*-*pgdS* | pHT43 derivate, P_grac_-SP_amyQ_-PgdS |  |
| pHT-*SP_aprE_*-*pgdS* | pHT43 derivate, P_grac_-SP_aprE_-PgdS |  |
|  |  |  |
|  |  |  |
| Strians |  |  |
| *E. coli* DH5α | F^–^ *endA1 glnV44 thi-1 recA1 relA1 gyrA96 deoR nupG* φ80d*lacZ*ΔM15  Δ(*lacZYA-argF*)*U169 hsdR17* (*r_K_*^–^ *m_K_*^–^) λ^–^ | Lab stock |
| *B. subtilis* 168 | *trpC2* |  |
| BS1 | *B. subtilis* 168 derivate, P_43_-*pgsBCAE* | This study |
| BS2 | *B. subtilis* 168 derivate, P_aprE_-*pgsBCAE* |  |
| BS3 | *B. subtilis* 168 derivate, P_xylA_-*pgsBCAE* |  |
| BS4 | *B. subtilis* 3 derivate, Δ*cwlO* |  |
| BS5 | *B. subtilis* 3 derivate, Δ*pgdS* |  |
| BS6 | *B. subtilis* 3 derivate, Δ*ggt* |  |
| BS7 | *B. subtilis* 3 derivate, Δ*cwlO*Δ*pgdS* |  |
| BS8 | *B. subtilis* 3 derivate, Δ*cwlO*Δ*ggt* |  |
| BS9 | *B. subtilis* 3 derivate, Δ*pgdS*Δ*ggt* |  |
| BS10 | *B. subtilis* 3 derivate, Δ*cwlO*Δ*pgdS*Δ*ggt* |  |
| BS11 | *B. subtilis* 10 carrying plasmid pHT-*SP_PgdS_*-*pgdS* |  |
| BS12 | *B. subtilis* 10 carrying plasmid pHT-*SP_amyQ_*-*pgdS* |  |
| BS13 | *B. subtilis* 10 carrying plasmid pHT-*SP_aprE_*-*pgdS* |  |
